# Changes in Antithrombotic Therapy Prescription in Patients with a Combination of Atrial Fibrillation and Myocardial Infarction in a Specialised Inpatient Department from 2016–2019 and Associations with Prognosis

**DOI:** 10.3390/medicina59091556

**Published:** 2023-08-27

**Authors:** Sergey Stepanovich Yakushin, Kristina Gennadievna Pereverzeva

**Affiliations:** Department of Hospital Therapy, Ryazan State Medical University, 390026 Ryazan, Russia

**Keywords:** atrial fibrillation, myocardial infarction, anticoagulant therapy, triple antithrombotic therapy, dual antiplatelet therapy, prognosis

## Abstract

*Background and Objectives*: The problem of treating patients with atrial fibrillation and myocardial infarction is relevant. The issue of optimal antithrombotic therapy in these patients has not been definitively resolved. This work analyzes the influence of clinical factors and treatment on the long-term prognosis of patients. *Materials and Methods*: The research included 360 patients with atrial fibrillation and myocardial infarction during 2016–2019. *Results*: The factors associated with fatal outcomes were age (hazard ratio (HR): 1.05; 95% confidence interval (CI): 1.03–1.07; *p* < 0.001); stroke (HR: 1.95; 95% CI: 1.27–3.00; *p* = 0.0002); glomerular filtration rate (HR: 0.988; 95% CI: 0.978–0.998; *p* = 0.03); left ventricular ejection fraction (HR: 0.975; 95% CI: 0.957–0.999; *p* = 0.007); and aspirin (HR: 0.48; 95% CI: 0.31–0.73; *p* < 0.001). The factors associated with the combined endpoint were chronic kidney disease (HR: 1.46; 95% CI: 1.01–2.10; *p* = 0.04); HAS-BLED (HR: 1.23; 95% CI: 1.06–1.43; *p* = 0.007); percutaneous coronary intervention (HR: 0.70; 95% CI: 0.51–0.96; *p* = 0.03); and aspirin (HR: 0.65; 95% CI: 0.44–0.97; *p* = 0.03). *Conclusions*: Double and triple antithrombotic therapy were not associated with outcomes. Aspirin improved the prognosis for survival and the combined endpoint.

## 1. Introduction

There is a two-way interconnection between atrial fibrillation (AF) and myocardial infarction (MI): MI can contribute to the onset of AF, and AF can lead to the development of MI [1,2]. Further, 14–18% of patients with AF have had MI in the past [2]. From 2.3% to 21% of patients with acute MI have constant or paroxysmal AF in past medical history [3,4]. Another 2–25% of patients without preceding AF will develop it during or after acute MI [3]. In concurrence with this, AF worsens the long-term prognosis of patients with MI and determines their higher mortality [5].

Now, clinical guidelines and consensus papers bring under regulation the necessity for patients to be prescribed antithrombotic therapy (ATT), which combines an antiaggregant(s) and anticoagulant, and to modify its duration depending on the risks of thrombosis and bleeding. We can trace the changes in the management of prescribing ATT in the period of 2016–2020 in the published clinical guidelines [6,7]. They are aimed at reducing the time of triple ATT (TATT) intake in order to increase its safety.

Thus, when analyzing the frequency of prescription TATT in real clinical practice earlier, the researchers found it insufficient, but now it seems to be excessive. Double ATT (antiaggregant and anticoagulant) according to clinical research compared with TATT in the long-term period is associated with a comparable number of ischemic strokes, recurrent MI, stent thrombosis, and fatal outcomes with a statistically significant decrease in the number of major/clinically significant bleeding [8,9,10]. Along with that, there are no similar data obtained in real clinical practice. We have analyzed the changes in the prescription of various combinations of ATT and its effect on long-term prognosis in patients with AF of nonvalvular etiology who were hospitalized in a cardiological in-patient department due to MI.

The aim is to assess the impact of clinical and anamnestic factors, as well as administered therapy, on the prognosis of patients with non-valvular atrial fibrillation who were hospitalized in a cardiological facility due to myocardial infarction between 2016 and 2019.

## 2. Materials and Methods

We conducted a retrospective study on the basis of the Ryazan Regional Clinical Cardiological Dispensary (Ryazan, Russia). The retrospective period of the study covered 1 January 2016–31 December 2019, and the prospective period covered January 2020–January 2021.

Inclusion Criteria:

- Age over 18;
- Hospitalization due to acute MI in the Ryazan Regional Clinical Cardiology Dispensary (Ryazan, Russia);
- An established diagnosis of acute MI based on the “third universal definition of myocardial infarction” and “fourth universal definition of myocardial infarction” (2018) [11,12];
- An established diagnosis of AF based on the Clinical Guidelines “2016 ESC Guidelines for the Management of Atrial Fibrillation developed in collaboration with EACTS” [6].

There were no exclusion criteria.

Case histories and telephone contact were the source of information about patients and their drug therapy. The participation in the retrospective analysis did not require any diagnostic/curative procedures that went beyond the clinical guidelines; the patients signed informed consent in accordance with the standard form for a medical institution. We received the consent of patients to participate in the prospective part of the study and to publish anonymous medical information for scientific purposes from all study participants orally by phone. The research was conducted in accordance with the Declaration of Helsinki and the research protocol was approved by the local ethical committee of the Federal State Budgetary Educational Institution of Higher Education at the “Ryazan State Medical University named after academician I.P. Pavlov” of the Ministry of Health of the Russian Federation on 10 December 2018 (protocol No. 5) [13].

We divided the patients into 2 groups depending on the time of admission: those who were admitted in the period from 1 January 2016 to 31 December 2017 and those who were admitted from 1 January 2018 to 31 December 2019. A total of 360 patients with MI and AF were included in the research: in 2016–2017, 104, and 2018–2019, 256 patients. We described these groups of patients in detail in a previously published paper [13,14]. We analyzed patients on the CHA_2_DS_2_-VASc scales (congestive heart failure, hypertension, age 75 and older, diabetes mellitus, stroke or transient ischemic attack, vascular diseases, age 65 to 74 years, women) and HAS-BLED (hypertension, impaired kidney/liver function, stroke, bleeding anamnesis or predisposition, labile international normalized ratio, old age, simultaneous drug/alcohol use) points [15,16].

At least 12 months after the index event (MI), we contacted patients or their relatives by phone to evaluate the patient’s vital status as well as to record the incidence of non-fatal MI, cerebral stroke (CS), and bleeding. In this study, we analyzed patient mortality and the combined endpoint (CEP), which includes fatal outcomes after the index event, non-fatal MI and CS, and fatal and non-fatal MI and CS separately. The prospective part of this work, analyzing the vital status of patients and the frequency of registration of MI and CS in patients hospitalized in 2018–2019, was described in a previously published article [14].

The statistical analysis of the material was carried out using the Statistica 11.0 program (Statsoft Inc., Tulsa, OK, USA), and MS Excel 2019 package (Microsoft, Redmond, WA, USA). The distribution of the obtained data was evaluated by the Shapiro–Wilk test. Quantitative features had a different distribution from normal and were described by the median, lower, and upper quartiles in the form of Me (Q1; Q3). The comparison of two unrelated groups on a qualitative level was carried out using the creation of contingency tables, using Pearson’s χ2, Yates’ χ2 correction and Fisher’s exact tests to examine hypotheses. The comparison of two unrelated groups on a quantitative basis was carried out using the Mann–Whitney test. A Cox proportional hazards regression model was used to analyze the outcomes. At first, we conducted a univariate analysis. The factors that were associated with the outcome were consistently included in the multivariable analysis. The multivariable analysis included those factors for which a relationship with the outcome was obtained in the single-factor analysis with *p* < 0.05. These factors were included in the regression model step by step. In the final regression model, factors with a *p*-value <0.05 were included. The analysis results are presented as the hazard ratios (HR) and the corresponding 95% confidence intervals (CI). Differences at *p* < 0.05 were considered to be statistically significant differences.

## 3. Results

### 3.1. Characteristics of the Sample

A total of 360 patients with MI and AF were included in the research: in 2016–2017, 104, and from 2018–2019, 256 patients. Table 1 provides a summary of the main baseline characteristics of the patients.

When evaluating the risk of thromboembolic complications on the CHA_2_DS_2_-VASc scale, the median scores for patients hospitalized in 2016–2017 and in 2018–2019 were the same and composed 5.0 (4.0; 6.0) points, *p* = 0.21.

We did not receive the changes in the incidence of individual risk factors of thromboembolic complications on the CHA_2_DS_2_-VASc scale in the 2016–2019 period.

When evaluating the risk of hemorrhagic complications while taking oral anticoagulants (OAC) on the HAS-BLED scale, the median score for patients hospitalized in 2016–2017 was 2.0 (2.0; 3.0) points; in 2018–2019, it also was composed of 2.0 (2.0; 2.0) points, *p* = 0.32.

When analyzing the degree of incidence of risk factors on the HAS-BLED scale in 2016–2017 and 2018–2019, the presence of bleeding and/or anemia in past medical history or at the present time turned out to be statistically significantly different factors: 2016–2017, in 1.9% (2) cases; 2018–2019, in 11.7% (30) cases, *p* = 0.003; and taking drugs that increase the risk of bleeding: in 2016–2017, 97.7% (101) cases; in 2018–2019, 100% (256) cases, *p* = 0.02.

### 3.2. ATT in Patients with AF and MI

Changes in the profile of prescribed ATT therapy for AF and MI in 2016–2019 are represented in Table 2.

In 2016–2017, among patients undergoing PCI (n = 55), 9.1% (5) administered TATT, 7.3% (4)—OAC in combination with one antiaggregant, and 83.6% (46)—DAPT.

In 2018–2019, among patients undergoing PCI (n = 145), 51.0% (74) administered TATT, 42.1% (61) DAPT, and 6.9% (10) combined OAC and antiaggregant.

**Figure 1:**
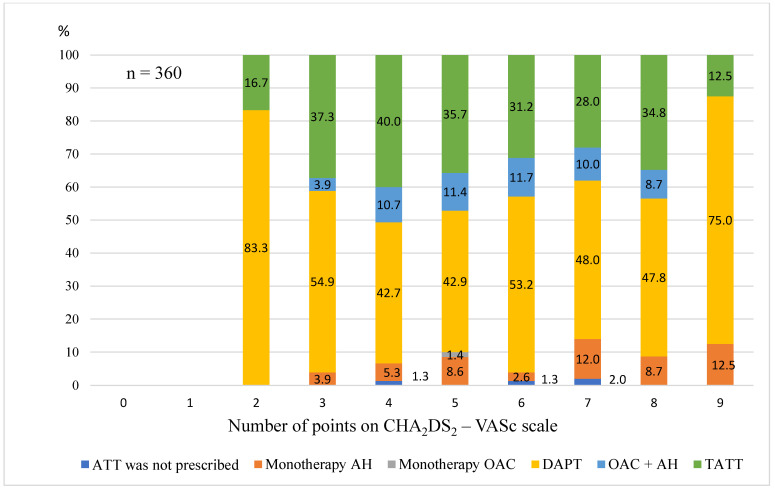
Composition of ATT in patients with atrial fibrillation and myocardial infarction in the period of 2016–2019; antiaggregant. Abbreviations: ATT, antithrombotic therapy; OAC, oral anticoagulants; DAPT, dual antiplatelet therapy; TATT, triple antithrombotic therapy.

The frequency of prescribing antiaggregant and OAC did not depend on the number of points on the CHA_2_DS_2_–VASc scale. Its distribution in patients with CHA_2_DS_2_–VASc scores of 2–9 is shown in Figure 1.

### 3.3. Concomitant Therapy in Patients with AF and MI

Concomitant drug therapy is presented in Table 3.

### 3.4. Factors Associated with an Increased Risk of Death and CEP in Patients with AF and MI

After 18 (13; 25) months from the moment of inclusion, 118 patients died, and 154 patients had at least one of the events combined in the CEP. The degree of incidence of clinical and anamnesis factors in the group of deceased and surviving patients and in the group of those who reached and did not reach CEP is represented in Table 4.

The frequency of prescribing ATT in the group of deceased and surviving patients and in the group of those who reached and did not reach the CEP is represented in Table 5.

The differences in concomitant therapy between the groups are presented in Table 6.

As a result of univariate analysis, factors associated with the risk of death were identified. They are presented in Table 7.

The results of the multivariable analysis are presented in Table 8.

As a result of univariate analysis, factors associated with the risk of CEP were identified. They are presented in Table 9.

The results of the multivariable analysis are presented in Table 10.

## 4. Discussion

In the research, the incidence of AF in MI was 12.5% and was in the frequency range of 2–23% received by other authors, both in other countries [17,18,19] and in other regions of Russia [20,21].

At the beginning of this research, the prescription rate of ATT was 6.7%, and it was higher than the prescription rate of ATT received in the 2013–2014 CAMI research in China, where it was 1.7% [22] and much lower than indicated in the research of Z. G. Tatarintseva et al. [23], conducted in Krasnodar Krai, where the frequency of TATT prescription in the period of 2015–2017 was 62.8%, and the work of M.V. Solovieva and S.A. Boldueva [21], conducted in the period of 2013–2018 in St. Petersburg, where this index was 80.5%.

Later on, in the course of the research in 2018–2019, an increase in the frequency of prescribing TATT to 44.9% was registered. Comparable frequency of TATT prescription during this period of time has been shown in a number of different researches. In particular, in the Romanian research of Cotoban AG et al. [24], where the frequency of TATT prescription was 52.6%. In the Chinese research by Mori H et al., the frequency was 44% [25]. And in the research by Suo N et al. [26], it was 30.5%.

The latest cited research is of particular interest. It was conducted in China and during it, the researchers analyzed the frequency of prescribing ATT in changes from 2017 to 2019 [25]. Despite the fact that the majority of patients were still prescribed DAPT without OAC at discharge, the percentage of DAPT decreased from 70% to <50% at 3 years, *p* < 0.001. In the research conducted in Ryazan, from 2016 to 2017 to 2018–2019, the frequency of DAPT decreased from 76.9% to 37.9%.

The frequency of prescribing combination therapy (OAC with one or two antiaggregant drugs) in the Chinese research increased from 27.2% to 50.0% over the same time period, and in the research conducted in Ryazan, it was from 16.3% to 54.7%.

At the same time, the percentage of patients who did not receive ATT remained the same in both studies [21].

However, despite the increase in the prescription of TATT in real clinical practice, data from clinical research published in 2020 and international guidelines and consensus documents recommend focusing on the fact that dual ATT (OAC + antiaggregant) seems to be better than TATT (OAC + DAPT), as there is a statistically significant reduction in the number of major/clinically significant bleeding, a similar number of ischemic strokes, a similar or non-significantly higher number of MI and stent thrombosis, the same number of cardiovascular events and identical overall mortality, and it is advisable to reduce the prescription of the duration of TATT in routine cases to 1 week. Only in those situations where the risks of ischemic events prevail over the risk of bleeding in patients with a low risk of the latter is it recommended to prolong therapy [6].

In concurrence with this, in all the analyzed case histories by us, in all cases of TATT prescription, no risks of ischemic events and/or bleeding were indicated, which allows us to suggest that, at least in some cases, TATT prescription was not associated with prevailing coronary risks and, in accordance with the latest clinical guidelines for the management of patients with AF [7], the volume of ATT in these patients would be excessive.

Data from other observational studies also confirm the information that in clinical practice, TATT is prescribed more often and without reason [27]. The prospective MATADOR-PCI registry in 2018–2019 included 598 patients with acute coronary syndrome receiving PCI and concomitant AF. Double antiplatelet therapy at discharge was prescribed for 26%, TAT for 65% and DAT for 9% of patients [28,29]. The most commonly used oral P2Y12 receptor inhibitor in this study was clopidogrel, while ticagrelor either or prasugrel was prescribed to 2.3% and 13.5% of patients [30]. In our study, doctors prescribed clopidogrel in 85.6% of cases and ticagrelor in 12.2%.

However, the received changes in ATT prescription for patients with AF and MI at that time, especially the increase in TATT prescription, should be regarded positively as a result of a long and gradual implementation of the ongoing clinical recommendations at that time (2016), in which is stated that “in patients with AF and atrial flutter at risk of stroke and acute coronary syndrome undergoing PCI, TATT is recommended to be extended up to 6 months after PCI” [4]. In the same recommendations, it is indicated that a high risk of bleeding is compared with the risk of recurrent acute coronary syndrome or stent thrombosis, and it is possible to reduce the duration of TATT to 1 month. Dual therapy of OAC with aspirin or clopidogrel may be considered in certain cases, especially for patients who have not undergone stenting [4]. A similar fact of gradual implementation of clinical recommendations is shown in other works [27,31]. In this regard, it is advisable to conduct active educational campaigns. They will help to quickly introduce new clinical recommendations into clinical practice [27].

As for the impact of the prescribed ATT therapy on the prognosis, it was not administered in this research, which may be due to the limited duration of observation, the small number of patients and the fact that in this research the frequency of prescription of ATT was analyzed and not the use of ATT. This rationale seems reasonable, especially due to the fact that similar research that analyzed the impact of ATT on the prognosis of patients with MI and AF were not administered “significant differences in the impact on one-year prognosis depending on the volume of ATT (*p* > 0.05 for all endpoints)” [22].

At the same time, aspirin improved the prognosis for survival and CEP.

However, the researchers evaluated the long-term prognosis for the entire period after discharge (mean 2.3 ± 1.9 years, maximum 7.2 years) when comparing the group of patients who did not take anticoagulants after discharge or administered them in the incorrect doses, patients who took anticoagulants adequately had significant differences in the onset of cardiovascular events compared to the patients who took anticoagulants inadequately. In case of incorrect intake or cancellation of anticoagulants, there was an increased risk of CS and a CEP: recurrent MI and CS and cardiovascular mortality [23].

## 5. Conclusions

The conducted retro-prospective research showed that the frequency of TATT at discharge from the hospital in 2018–2019 increased by 6.7 times compared to 2016–2017 and was 44.9%. The frequency of prescription of oral anticoagulants in 2018–2019 also increased by 3.4 times compared to 2016–2017 and was 54.7%, but currently, it is insufficient.

The factors increasing the risk of fatal outcomes were: older age and stroke. The factors enhancing survival rate were higher glomerular filtration rate, higher left ventricular ejection fraction and aspirin intake.

The factors increasing the risk of CEP were chronic kidney disease and a higher HAS-BLED score. The factors reducing the risk of CEP were PCI and aspirin intake.

Research limitations: This research was retro prospective, had a short observation period and a small number of patients, and analyzed only the frequency of prescription and not the use of antithrombotic therapy.

## Figures and Tables

**Table 1:** Patients’ characteristics.

Value	All Patients	2016–2017	2018–2019	*p* Value
N	360	104	256	
Female sex, *n* (%)	157 (43.6)	44 (44.4)	113 (44.2)	0.75
Age, years Me (Q1; Q3)	70.0 (63.0; 78.0)	70.0 (61.0; 78.0)	71.0 (65.0; 79.3)	0.09
Age 64–75 years, *n* (%)	116 (32.2)	28 (26.9)	88 (34.4)	0.17
Age > 75 years, *n* (%)	141 (39.2)	40 (38.5)	101 (39.5)	0.86
BMI, kg/m^2^ (Me (Q1; Q3))	28.4 (24.9; 31.1)	28.9 (24.9; 32.1)	27.8 (25.8; 31.1)	0.81
AF type				
Paroxysmal, *n* (%)	196 (54.4)	64 (61.5)	132 (51.6)	0.08
Persistent, *n* (%)	87 (24.2)	20 (19.2)	67(26.2)	0.16
Permanent, *n* (%)	77 (21.4)	20 (19.2)	57(22.3)	0.52
Comorbidities				
Hypertension, *n* (%)	349 (96.9)	98 (94.2)	251 (98.0)	0.06
Stroke or transient ischemic attack, *n* (%)	54 (15.0)	17 (16.3)	37 (14.5)	0.65
Old myocardial infarction, *n* (%)	37 (10.3)	14 (13.4)	23 (9.0)	0.2
Prior PCI, *n* (%)	20 (5.6)	8 (7.7)	12 (4.7)	0.26
Diabetes mellitus, *n* (%)	109 (30.3)	28 (26.9)	81 (31.6)	0.43
Chronic heart failure, *n* (%)	360 (100.0)	104 (100.0)	256 (100.0)	-
LVEF, % Me (Q1; Q3)	51.0 (44.0; 58.0)	51.0 (44.0; 57.0)	51.0 (44.0; 58.0)	0.63
LVEF < 40%, %	48 (13.3)	17 (16.3)	31 (12.1)	0.34
Chronic kidney disease, *n* (%)	80 (22.2)	19 (18.3)	61 (23.4)	0.25
Impaired renal function, *n* (%)	18 (5.0)	7 (6.7)	11 (4.3)	0.49
Vascular disease (e.g., PAD), *n* (%)	5 (1.4)	1 (1.0)	4 (1.6)	0.66
Chronic liver disease, *n* (%)	17 (4.7)	9 (8.5)	8 (3.1)	0.05
Bleeding/Anemia in the past or currently, *n* (%)	32 (8.9)	2 (1.9)	30 (11.7)	0.001 *
Active cancer, *n* (%)	13 (3.6)	4 (3.8)	9 (3.5)	0.88
Alcohol use disorder, *n* (%)	0 (0)	0 (0)	0 (0)	–
Admission of MR that increases the risk of bleeding, *n* (%)	357 (99.2)	101 (97.7)	256 (100.0)	0.02 *
Type of index MI				
STEMI, *n* (%)	280 (77.7)	82 (78.8)	198 (77.3)	0.76
NSTEMI, *n* (%)	80 (22.3)	22 (21.2)	58 (22.7)	
Management of index MI				
PCI, *n* (%)	200 (55.5)	55 (52.9)	145 (56.6)	0.51
Medical, *n* (%)	160 (44.5)	49 (47.1)	111 (43.4)	
Objective data				
Heart rate, b.p.m. (Me (Q1; Q3))	78.0 (70.0; 88.0)	78.0 (67.5; 82.3)	78.0 (70.0; 88.0))	0.68
SBP, mm Hg (Me (Q1; Q3))	140.0 (120.0; 150.0)	140.0 (120.0; 150.0)	140.0 (120.0; 150.0)	0.83
DBP, mm Hg (Me (Q1; Q3))	80.0 (80.0; 90.0)	80.0 (71.5; 90.0)	80.0 (80.0; 90.0)	0.09
Laboratory results (Me (Q1; Q3))				
Serum creatinine, μmol/L	103.1 (89.6; 127.0)	111.0 (93.8; 131.5)	102.0 (87.3; 123.1)	0.25
GFR, ml/min/1.72 m^2^	54.4 (43.5; 68.9)	54,2 (42.4; 68.5)	54.4 (43.6; 69.1)	0.59
Total cholesterol, mmol/L	4.9 (4.2; 5.8)	4.9 (4.2; 5.9)	4.9 (4.2; 5.4)	0.92
LDL cholesterol, mmol/L	2.9 (2.2; 3.8)	2.8 (2.2; 3.7)	2.7 (2.4; 4.1)	1.00
HDL cholesterol, mmol/L	1.1 (0.9; 1.3)	1.1 (0.9; 1.3)	1.1 (0.9; 1.2)	0.72
Triglycerides, mmol/L	1.3 (1.0; 1.9)	1.3 (1.0; 2.0)	1.4 (1.2; 2.6)	0.31
Fasting glucose, mmol/L	5.1 (4.6; 6.6)	5.3 (4.6; 6.6)	4.8 (4.2; 6.4)	0.10
Risk scores				
CHA_2_DS_2_-VASc score	5.0 (4.0; 6.0)	5.0 (4.0; 6.0)	5.0 (4.0; 6.0)	0.21
HAS-BLED score	2.0 (2.0; 2.0)	2.0 (2.0; 3.0)	2.0 (2.0; 2.0)	0.32

Abbreviations: BMI, body mass index; AF, atrial fibrillation; PCI, percutaneous coronary intervention; LVEF, left ventricular ejection fraction; PAD, peripheral artery diseases; STEMI, ST-segment elevation myocardial infarction; NSTEMI, non-ST-segment elevation myocardial infarction; MI, myocardial infarction; b.p.m., beats per minute; SBP, systolic blood pressure; DBP, diastolic blood pressure; GFR, glomerular filtration rate to the Chronic Kidney Disease Epidemiology Collaboration; LDL, low-density lipoproteins; HDL, high-density lipoproteins; * *p* < 0.05 was considered statistically significant.

**Table 2:** ATT.

Value N	All Patients	2016–2017	2018–2019	*p* Value
	360	104	256	
Total OAC, *n* (%)	157 (43.6)	17 (16.3)	140 (54.7)	<0.001 *
Vitamin K antagonist, *n* (%)	62 (17.2)	11 (10.6)	51 (19.9)	0.03 *
DOAC, *n* (%)	95 (26.4)	6 (5.8)	89 (34.8)	<0.001 *
Apixaban, *n* (%)	0 (0)	0 (0)	0 (0)	-
Dabigatran, *n* (%)	1 (0.3)	0 (0)	1 (0.3)	-
Rivaroxaban, *n* (%)	94 (26.1)	6 (5.8)	88 (34.4)	<0.001 *
TATT, *n* (%)	122 (33.9)	7 (6.7)	115 (44.9)	<0.001 *
OAC + antiaggregant, *n* (%)	34 (9.4)	9 (8.7)	25 (9.8)	0.74
DAPT, *n* (%)	177 (49.2)	80 (76.9)	97(37.9)	<0.001 *
Acetylsalicylic acid, *n* (%)	303 (84.2)	91 (85.7)	212 (82.8)	0.27
Clopidogrel, *n* (%)	308 (85.6)	79 (76.0)	229 (89.8)	0.001 *
Ticagrelor, *n* (%)	44 (12.2)	17 (16.3)	27 (10.6)	0.12
Antiaggregant (monotherapy), *n* (%)	23 (6.4)	4 (3.8)	19 (7.4)	0.15
Monotherapy OAC, *n* (%)	1 (0.3)	1 (1.0)	0 (0)	–
ATT was not prescribed, *n* (%)	3 (0.8)	3 (2.9)	0 (0)	–

Abbreviation: OAC, oral anticoagulants; DOAC, direct oral anticoagulants; TATT, triple antithrombotic therapy; ATT, antithrombotic therapy; DAPT, dual antiplatelet therapy; * *p* < 0.05 was considered statistically significant.

**Table 3:** Concomitant drug therapy.

Value	All Patients	2016–2017	2018–2019	*p* Value
N	360	104	256	
Amiodarone, *n* (%)	193 (53.6)	61 (58.7)	132 (51.6)	0.22
Angiotensin-converting enzyme inhibitors, *n* (%)	331 (91.9)	92 (88.5)	239 (93.4)	0.13
Angiotensin receptor blockers, *n* (%)	15 (4.2)	7 (6.7)	8 (3.1)	0.11
Beta-blockers, *n* (%)	194 (53.9)	63 (60.6)	131 (51.2)	0.11
Aldosterone blockers, *n* (%)	86 (23.9)	21 (20.2)	65 (25.4)	0.29
Diuretics, *n* (%)	140 (38.9)	43 (41.3)	97 (37.9)	0.54
Statins, *n* (%)	352 (97.8)	99 (95.2)	253 (98.8)	0.11

**Table 4:** Clinical and anamnesis factors in the group of deceased and surviving patients and in the group of those who reached and did not reach the CEP.

Value	Deceased (n = 118)/Surviving (n = 239)	*p* Value	Reached CEP (n = 154)/Did Not Reach CEP (n = 203)	*p* Value
Age, years (Me (Q1; Q3))	77.0 (70.0; 81.0)/68.0 (61.0; 76.0)	<0.001 *	74.0 (68.0; 81.0)/68.0 (61.0; 76.0)	<0.001 *
Age 64–75 years, *n* (%)	38 (32.2)/78 (32.6)	0.93	51 (33.1)/65 (32.0)	0.83
Age ≥ 75 years, *n* (%)	68 (57.6)/73 (30.5)	<0.001 *	77 (50.0)/64 (31.5)	0.0004 *
Male sex, *n* (%)	57 (48.3)/143 (59.8)	0.04 *	78 (50.6)/122 (60.1)	0.07
AF type				
Paroxysmal, *n* (%)	66 (55.9)/130 (54.4)	0.78	92 (59.7)/104 (51.2)	0.11
Persistent, *n* (%)	29 (24.6)/57 (23.8)	0.88	34 (22.1)/52 (25.6)	0.53
Permanent, *n* (%)	23 (19.5)/52 (21.8)	0.62	28 (18.2)/47(23.2)	0.25
Comorbidities				
Hypertension, *n* (%)	112 (94.9)/234 (97.9)	0.19	146 (94.8)/200 (98.5)	0.06
Stroke or transient ischemic attack, *n* (%)	35 (29.7)/15 (6.3)	<0.001 *	38 (24.7)/12 (5.9)	<0.001 *
Old MI, *n* (%)	12 (10.2)/23 (9.6)	0.87	14 (9.1)/21 (10.3)	0.69
Prior PCI, *n* (%)	7 (5.9)/11 (4.6)	0.59	7 (4.5)/11 (5.4)	0.71
Diabetes mellitus, *n* (%)	38 (32.2)/71 (29.7)	0.63	48 (31.2)/61 (30.0)	0.82
Chronic heart failure, n (%)	118 (100.0)/239 (100.0)	-	154 (100.0)/203 (100.0)	-
LVEF, % (Me (Q1; Q3))	48.0 (41.0; 56.0)/52.0 (45.0; 59.0)	0.01 *	49.0 (42.0; 56.0)/52.0 (45.0; 59.0)	0.02 *
LVEF < 40%, %	21 (17.8)/27 (11.3)	0.09	27 (17.5)/21 (10.3)	0.04 *
Chronic kidney disease, *n* (%)	43 (36.4)/37 (15.2)	<0.001 *	49 (31.8)/31 (15.3)	<0.001 *
Vascular disease (e.g., PAD), *n* (%)	1 (0.8)/4 (1.7)	0.47	1 (0.6)/4 (2.0)	0.39
Chronic liver disease, *n* (%)	9 (7.6)/8 (3.3)	0.07	11 (7.1)/6 (3.0)	0.06
Bleeding/anemia in the past or currently, n (%)	16 (13.6)/16 (6.7)	0.03 *	20 (13.0)/12 (6.0)	0.02 *
Active cancer, *n* (%)	7 (5.9)/6 (2.5)	0.10	7 (4.5)/6 (3.0)	0.31
Alcohol use disorder, *n* (%)	0 (0)	-	0 (0)	-
Admission of MR that increases the risk of bleeding, *n* (%)	117 (99.2)/238 (99.6)	0.61	152 (98.7)/203 (100)	
Type of index MI				
STEMI, *n* (%)	94 (79.7)/183 (76.6)	0.51	121 (78.6)/156 (76.8)	0.62
NSTEMI, *n* (%)	24 (20.3)/56 (23.4)		33 (21.4)/47 (23.2)	
Management of index MI				
PCI, *n* (%)	53 (44.9)/152 (63.6)	<0.001 *	75 (48.7)/130 (64.0)	0.004 *
Medical, *n* (%)	65 (55.1)/87 (36.4)		79 (51.3)/70 (34.5)	
Objective data				
Heart rate, b.p.m. (Me (Q1; Q3))	75.0 (66.0; 88.0)/78.0 (70.0; 90.0)	0.33	75.0 (69.5; 84.3)/78.0 (70.0; 90.0)	0.11
SBP, mm Hg (Me (Q1; Q3))	140.0 (122.5; 150.0)/136.0 (121.0; 150.0)	0.91	132.5 (122.0;150.0)/140.0 (121.0; 150.0)	0.99
DBP, mm Hg (Me (Q1; Q3))	80.0 (72.8; 90.0)/80.0 (80.0; 90.0)	0.09	80.0 (80.0; 90.0)/80.0 (80.0; 90.0)	0.66
Laboratory results (Me (Q1; Q3))				
Serum creatinine, μmol/L	117.0 (99.2; 138.8)/100.0 (85.0; 119.0)	<0.001 *	115.5 (93.0; 136.0)/101.0 (88.0; 119.0)	<0.001 *
GFR, (Q1; Q3), ml/min/1.72 m^2^	47.8 (35.3; 63.2)/60.1 (45.6; 76.6)	<0.001 *	51.3 (37.0; 65.1)/60.0 (45.6; 73.6)	<0.001 *
Total cholesterol, mmol/L	4.9 (4.2;5.7)/5.0 (4.2;5.9)	0.61	4.9 (4.2;5.7)/5.0 (4.2;5.9)	0.61
LDL cholesterol, mmol/L	3.5 (3.0; 4.3)/3.1 (2.4; 4.1)	0.54	3.5 (3.0; 4.4)/3.1 (2.4; 4.1)	0.53
HDL cholesterol, mmol/L	1.1 (0.9; 1.3)/1.1 (0.9; 1.2)	0.71	1.1 (0.9; 1.3)/1.1 (0.9; 1.2)	0.71
Triglycerides, mmol/L	1.4 (1.0; 2.2)/1.5 (1.2; 2.2)	0.58	1.4 (1.0; 2.2)/1.6 (1.2; 2.4)	0.39
Fasting glucose, mmol/L	5.7 (4.7;6.7)/5.6 (5.1; 6.1)	0.95	5.7 (4.6;6.0)/5.6 (5.1; 6.1)	0.92
Risk scores				
CHA_2_DS_2_-VASc score	6.0 (4.0; 7.0)/4.0 (3.0; 6.0)	<0.001 *	4.0 (3.0;6.0)/4.0 (3.0; 6.0)	0.24
HAS-BLED score	2.0 (2.0;3.0)/2.0 (1.3;2.0)	<0.001 *	2.0 (2.0;3.0)/2.0 (1.0;2.0)	<0.001 *

Abbreviation: CEP, combined endpoint; BMI, body mass index; AF, atrial fibrillation; PCI, percutaneous coronary intervention; LVEF, left ventricular ejection fraction; PAD, peripheral artery diseases; MR, medicinal products; STEMI, ST-segment elevation myocardial infarction; NSTEMI, non-ST-segment elevation myocardial infarction; MI, myocardial infarction; b.p.m., beats per minute; SBP, systolic blood pressure; DBP, diastolic blood pressure; GFR, glomerular filtration rate to Chronic Kidney Disease Epidemiology Collaboration; LDL, low-density lipoproteins; HDL, high-density lipoproteins; * *p* < 0.05 was considered statistically significant.

**Table 5:** ATT in the group of deceased and surviving patients and in the group of those who reached and did not reach the CEP.

Value	Deceased (n = 118)/Surviving (n = 239)	*p* Value	Reached CEP (n = 154)/Did Not Reach CEP (n = 203)	*p* Value
OAC, *n* (%)	51 (43.2)/106 (44.4)	0.83	68 (44.2)/89 (43.8)	0.95
TATT, *n* (%)	32 (27.1)/90 (37.7)	0.048 *	43 (27.9)/79 (38.9)	0.03 *
Vitamin K antagonist, *n* (%)	17 (14.4)/45 (18.2)	0.30	25 (16.2)/37 (18.2)	0.44
DOAC, *n* (%)	34 (28.8)/61 (25.5)	0.53	43 (27.9)/52 (25.6)	0.65
Apixaban, *n* (%)	0 (0)/0 (0)	-	0 (0)/0 (0)	-
Dabigatran, *n* (%)	0 (0)/1 (0.4)	-	0 (0)/1 (0.5)	-
Rivaroxaban, *n* (%)	34 (28.8)/60 (25.1)	0.52	43 (27.9)/51 (25.1)	0.55
OAC + antiaggregant, *n* (%)	14 (9.3)/20 (7.9)	0.30	20 (13.0)/14 (6.9)	0.36
DAPT, *n* (%)	53 (44.9)/121 (50.6)	0.31	69 (44.8)/105 (51.7)	0.19
Acetylsalicylic acid, *n* (%)	89 (75.4)/214 (89.5)	<0.001 *	112 (73.2)/191 (94.1)	<0.001 *
Clopidogrel, *n* (%)	101(85.6)/207 (86.6)	0.79	134 (87.6)/174 (85.7)	0.61
Ticagrelor, *n* (%)	9 (7.6)/35 (14.6)	0.08	12 (7.8)/32 (15.8)	0.07
Antiaggregant monotherapy, *n* (%)	15 (33.4)/8 (3.4)	0.002 *	18 (11.7)/5 (2.5)	<0.001 *
Monotherapy OAC, *n* (%)	1 (3.7)/0 (0)	–	1 (0.6)/0 (0)	–
Absence of therapy, *n* (%)	3 (2.5)/0 (0)	–	3 (1.9)/0 (0)	–

Abbreviations: CEP, combined endpoint; OAC, oral anticoagulants; DOAC, direct oral anticoagulants; TATT, triple antithrombotic therapy; ATT, antithrombotic therapy; DAPT dual antiplatelet therapy; * *p* < 0.05 was considered statistically significant.

**Table 6:** Concomitant therapy in the group of deceased and surviving patients and in the group of those who reached and did not reach the CEP.

Value	Deceased (n = 118)/ Surviving (n = 239)	*p* Value	Reached CEP (n = 154)/Did Not Reach CEP (n = 203)	*p* Value
Amiodarone, *n* (%)	57 (48.3)/136 (56.9)	0.13	85 (55.2)/108 (52.7)	0.71
Angiotensin-converting Enzyme Inhibitors, *n* (%)	110 (93.2)/221 (92.4)	0.8	137 (89.0)/194 (95.6)	0.02 *
Angiotensin receptor blockers, *n* (%)	3 (2.5)/12 (5.0)	0.21	4 (2.6)/11 (5.4)	0.15
Beta-blockers, *n* (%)	66 (55.9)/128 (51.0)	0.37	77 (50.0)/117 (57.6)	0.15
Aldosterone blockers, *n* (%)	26 (22.0)/60 (25.1)	0.52	31 (20.1)/55 (27.1)	0.13
Diuretics, *n* (%)	55 (46.6)/88 (36.8)	0.08	71 (46.1)/69 (34.0)	0.02 *
Statins, *n* (%)	117 (99.2)/235 (98.3)	0.46	149 (96.8)/202 (99.5)	0.06

Abbreviation: CEP, combined endpoint; * *p* < 0.05 was considered statistically significant.

**Table 7:** Univariate analysis of death predictors.

Value	Unadjusted		Adjusted	
	Hazard Ratios (95% CI)	*p* Value	Hazard Ratios (95% CI)	*p* Value
Age, years	1.06 (1.04–1.09)	<0.001 *	1.06 (1.04–1.09)	<0.001 *
Age ≥ 75 years	2.4 (1.64–3.43)	<0.001 *	2.4 (1.64–3.43)	<0.001 *
Female sex	0.85 (0.59–1.23)	0.39	0.85 (0.59–1.23)	0.39
Stroke or transient ischemic attack	2.70 (1.80–4.01)	<0.001 *	2.70 (1.80–4.01)	<0.001 *
LVEF, %	0.965 (0.947–0.982)	<0.001 *	0.965 (0.947–0.982)	<0.001 *
Chronic kidney disease	2.24 (1.54–3.27)	<0.001 *	2.24 (1.54–3.27)	<0.001 *
Serum creatinine, μmol/L	1.006 (1.003–1.009)	<0.001 *	1.006 (1.003–1.009)	<0.001 *
GFR, mL/min/1.72 m^2^	0.974 (0.965–0.983)	<0.001*	0.974 (0.965–0.983)	<0.001 *
Bleeding/anemia in the past or currently	0.65 (0.33–1.29)	0.22	0.65 (0.33–1.29)	0.22
PCI	0.65 (0.45–0.93)	0.02 *	0.65 (0.45–0.93)	0.02 *
CHA_2_DS_2_-VASc score	1.35 (1.20–1.51)	<0.001 *	1.35 (1.20–1.51)	<0.001 *
HAS-BLED score	1.49 (1.28–1.74)	<0.001 *	1.49 (1.28–1.74)	<0.001 *
TATT	0.63 (0.39–1.02)	0.06	0.63 (0.39–1.02)	0.06
Acetylsalicylic acid	0.43 (0.25–0.75)	0.003 *	0.43 (0.25–0.75)	0.003 *
Antiaggregant monotherapy	1.29 (0.67–2.46)	0.49	1.29 (0.67–2.46)	0.49

Abbreviations: CI, 95% confidence intervals; PCI, percutaneous coronary intervention; LVEF, left ventricular ejection fraction; GFR, glomerular filtration rate to Chronic Kidney Disease Epidemiology Collaboration; TATT, triple antithrombotic therapy; * *p* < 0.05 was considered statistically significant.

**Table 8:** Multivariable analysis of death predictors.

Value	Unadjusted		Adjusted	
	Hazard Ratios (95% CI)	*p* Value	Hazard Ratios (95% CI)	*p* Value
Age, years	1.06 (1.04–1.08)	<0.001 *	1.05 (1.03–1.07)	<0.001 *
Stroke or transient ischemic attack	2.68 (1.80–3.98)	<0.001 *	1.95 (1.27–3.00)	0.002 *
GFR, mL/min/1.72 m^2^	0.974 (0.965–0.984)	<0.001 *	0.988 (0.978–0.998)	0.03 *
LVEF, %	0.965 (0.947–0.982)	<0.001 *	0.975 (0.957–0.999)	0.007 *
Acetylsalicylic acid	0.53 (0.35–0.80)	0.003 *	0.48 (0.31–0.73)	<0.001 *

Abbreviations: CI, 95% confidence intervals; LVEF, left ventricular ejection fraction; GFR, glomerular filtration rate to Chronic Kidney Disease Epidemiology Collaboration; * *p* < 0.05 was considered statistically significant.

**Table 9:** Univariate analysis of CEP predictors.

Value	Unadjusted		Adjusted	
	Hazard Ratios (95% CI)	*p* Value	Hazard Ratios (95% CI)	*p* Value
Age, years	1.02 (1.01–1.04)	0.006 *	1.02 (1.01–1.04)	0.006 *
Age ≥ 75 years	1.16 (0.83–1.60)	0.39	1.16 (0.83–1.60)	0.39
Female sex	0.91 (0.66–1.26)	0.59	0.91 (0.66–1.26)	0.59
Stroke or transient ischemic attack	1.49 (0.99–2.23)	0.06	1.49 (0.99–2.23)	0.06
LVEF, %	0.987 (0.971–1.003)	0.12	0.987 (0.971–1.003)	0.12
LVEF < 40%, %	1.35 (0.88–2.07)	0.18	1.35 (0.88–2.07)	0.18
Chronic kidney disease	1.67 (1.17–2.39)	0.004 *	1.67 (1.17–2.39)	0.004 *
Serum creatinine, μmol/L	1.003 (1.000–1.006)	0.088	1.003 (1.000–1.006)	0.09
GFR, mL/min/1.72 m^2^	0.989 (0.981–0.997)	0.009 *	0.989 (0.981–0.997)	0.009 *
Bleeding/anemia in the past or currently	1.53 (0.98–2.4)	0.062	1.53 (0.98–2.4)	0.06
PCI	0.68 (0.50–0.93)	0.017 *	0.68 (0.50–0.93)	0.017 *
CHA_2_DS_2_-VASc score	1.09 (0.98–1.21)	0.107	1.09 (0.98–1.21)	0.11
HAS-BLED score	1.27 (1.10–1.47)	0.001 *	1.27 (1.10–1.47)	0.001 *
Angiotensin-converting enzyme inhibitors	0.62 (0.37–1.04)	0.072	0.62 (0.37–1.04)	0.07
Diuretics	1.03 (0.74–1.44)	0.869	1.03 (0.74–1.44)	0.87
TATT	0.80 (0.56–1.14)	0.214	0.80 (0.56–1.14)	0.21
Acetylsalicylic acid	0.66 (0.45–0.97)	0.036 *	0.66 (0.45–0.97)	0.036 *
Antiaggregant monotherapy	1.30 (0.73–2.30)	0.375	1.30 (0.73–2.30)	0.38

Abbreviations: CEP, combined endpoint; LVEF, left ventricular ejection fraction; GFR, glomerular filtration rate to Chronic Kidney Disease Epidemiology Collaboration; * *p* < 0.05 was considered statistically significant.

**Table 10:** Multivariable analysis of CEP predictors.

Value	Unadjusted		Adjusted	
	Hazard Ratios (95% CI)	*p* Value	Hazard Ratios (95% CI)	*p* Value
Chronic kidney disease	1.68 (1.18–2.39)	0.004 *	1.46 (1.01–2.10)	0.044 *
HAS-BLED score	1.28 (1.10–1.48)	<0.001 *	1.23 (1.06–1.43)	0.007 *
PCI	0.66 (0.48–0.91)	0.011 *	0.70 (0.51–0.96)	0.028 *
Acetylsalicylic acid	0.65 (0.44–0.96)	0.029 *	0.65 (0.44–0.97)	0.033 *

Abbreviation: CI, 95% confidence intervals; PCI, percutaneous coronary intervention; * *p* < 0.05 was considered statistically significant.

## Data Availability

The datasets generated and analyzed during the study are available from the corresponding author upon reasonable request.
